# Transcriptome Sequencing Unravels Potential Biomarkers at Different Stages of Cerebral Ischemic Stroke

**DOI:** 10.3389/fgene.2019.00814

**Published:** 2019-09-24

**Authors:** You Cai, Yufen Zhang, Xiao Ke, Yu Guo, Chengye Yao, Na Tang, Pei Pang, Gangcai Xie, Li Fang, Zhe Zhang, Jincheng Li, Yixian Fan, Ximiao He, Ruojian Wen, Lei Pei, Youming Lu

**Affiliations:** ^1^Department of Pathology and Pathophysiology, School of Basic Medicine and Tongji Medical College, Huazhong University of Science and Technology, Wuhan, China; ^2^The Institute for Brain Research (IBR), Collaborative Innovation Center for Brain Science, Huazhong University of Science and Technology, Wuhan, China; ^3^Department of Neurobiology, School of Basic Medicine and Tongji Medical College, Huazhong University of Science and Technology, Wuhan, China; ^4^Wuhan Children’s Hospital (Wuhan Maternal and Child Healthcare Hospital), Tongji College of Medicine, Huazhong University of Science & Technology, Wuhan, China; ^5^Department of Neurology, Union Hospital, Tongji College of Medicine, Huazhong University of Science and Technology, Wuhan, China; ^6^Department of Pathology, Maternal and Child Health Hospital of Hubei Province, Wuhan, China; ^7^Medical School, Institute of Reproductive Medicine, Nantong University, Nantong, China; ^8^Raymond G. Perelman Center for Cellular and Molecular Therapeutics, Children’s Hospital of Philadelphia, Philadelphia, PA, United States; ^9^Department of Physiology, School of Basic Medicine and Tongji Medical College, Huazhong University of Science and Technology, Wuhan, China; ^10^Department of Physiology, School of Medicine, Jianghan University, Wuhan, China

**Keywords:** ischemic stroke, RNA-Seq, small RNA-Seq, microRNA, biomarker

## Abstract

Ischemic stroke, which accounts for 87% of all strokes, constitutes the leading cause of morbidity and mortality in China. Although the genetics and epigenetics of stroke have been extensively investigated, few studies have examined their relationships at different stages of stroke. This study assessed the characteristics of transcriptome changes at different stages of ischemic stroke using a mouse model of transient middle cerebral artery occlusion (tMCAO) and bioinformatics analyses. Cerebral cortex tissues from tMCAO mice at days 1, 3, 7, 14, and 28 were removed for RNA-Seq and small RNA-Seq library construction, sequencing, and bioinformatics analysis. We identified differentially expressed (DE) genes and miRNAs and revealed an association of the up-regulated or down-regulated DEmiRNAs with the correspondingly altered DEgene targets at each time point. In addition, different biological pathways were activated at different time points; thus, three groups of miRNAs were verified that may represent potential clinical biomarkers corresponding to days 1, 3, and 7 after ischemic stroke. Notably, this represents the first functional association of some of these miRNAs with stroke, e.g., miR-2137, miR-874-5p, and miR-5099. Together, our findings lay the foundation for the transition from a single-point, single-drug stroke treatment approach to multiple-time-point multi-drug combination therapies.

## Introduction

Ischemic stroke, which accounts for 87% of all strokes, constitutes the leading cause of morbidity and mortality in China (Wang et al., 2017). The pathophysiology of stroke is a complex and multifaceted process including excitotoxicity, inflammation, oxidative damage, ionic imbalance, apoptosis, angiogenesis, and neuroprotection (Deb et al., 2010). The current gold standard in acute ischemic stroke therapy is intravenous thrombolysis by administration of recombinant tissue plasminogen activator; however, this intervention has a very limited therapeutic window of only 3 h (van der Worp and van Gijn, 2007). Thus, more effective management and preventive strategies for stroke are highly anticipated (Feigin et al., 2015). For example, there is an urgent need for the identification of more effective stroke therapies along with molecular targets, and to elucidate the mechanism of ischemic stroke-induced brain damage (Nagaoka et al., 1976; Jeyaseelan et al., 2008; Stinear, 2017).

Notably, with the increase in the deposit of sequencing data into various public databases, bioinformatics analyses have the potential to relate alterations of dynamic gene expression networks to human diseases. RNA-Seq and Next-Generation Sequencing technology provide useful tools in the study of differentially expressed (DE) transcriptomes in disease vs. non-disease states or in various stages after ischemic stroke. However, as these analyses generate many DEgenes, effective bioinformatics analyses are key to sorting out genes that are involved in disease development and progression. Moreover, the number of positive target molecules will be low if only single-omics data are used (Sun and Hu, 2016); thus, multi-omics data may markedly improve the design of these studies. For example, a previous study combined high-throughput data with low-throughput data generated by routine cellular and molecular methods to more accurately identify target molecules (Li and Biggin, 2015).

MicroRNAs (miRNAs) comprise a class of small, conserved non-coding RNAs that post-transcriptionally regulate the expression of protein-coding genes primarily by binding to the 3′-untranslated region to inhibit translation or promote mRNA degradation (Wu et al., 2012). Each miRNA may be capable of targeting hundreds of protein-coding genes, depending on the cell context, and to date, it is believed that up to 50% of protein-coding genes in mammals are regulated by miRNAs (Krol et al., 2010). MiRNAs are expressed temporally and spatially in the brain to regulate synaptic plasticity, neuronal differentiation, and development (Fineberg et al., 2009). However, aberrant expression of miRNAs occurs in various diseases of the central nervous system such as stroke (Jeyaseelan et al., 2008; Dharap et al., 2009), Parkinson’s disease, Down’s syndrome, Alzheimer’s disease, and schizophrenia (Beveridge et al., 2008; Arena et al., 2017; Chen et al., 2017). Previous studies have also shown that miRNAs play an important role in stroke and regulate the pathophysiological processes thereof, highlighting their potential therapeutic importance in the development and progression of post-stroke depression (Yan et al., 2013), angiogenesis, remyelination (Wang et al., 2013), neurogenesis (Liu F. J. et al., 2013), and self-repair of the brain tissues (Liu X. S. et al., 2013). Furthermore, it has been reported that miRNAs could be useful as diagnostic biomarkers and therapeutic targets in various human diseases (Broderick and Zamore, 2011; Tan et al., 2011; van Rooij et al., 2012). To ascertain their utility for these purposes in stroke, in this study, we utilized a mouse model of transient middle cerebral artery occlusion (tMCAO) to assess differential gene expression at different stages of ischemic stroke using Next-Generation Sequencing technology to identify DE genes and miRNAs at multiple time points after stroke. Bioinformatics analysis revealed that discrete clusters of miRNAs could be identified as potential clinical biomarkers corresponding to distinct periods after ischemic stroke, laying the foundation for multiple time point multi-drug combination therapies to more effectively counter the detrimental effects of this disease.

## Materials and Methods

### Animals

This study was approved by the Institutional Animal Care and Use Committee of Huazhong University of Science and Technology (Wuhan, China) and followed the Guidelines of the Care and Use of Laboratory Animals issued by the Chinese Council on Animal Research. Adult male C57BL/6J mice were purchased from the National Resource Center of Model Mice (Nanjing, China) and housed and bred in groups of 3–5 mice *per* cage under a 12-h light-dark cycle and consistent ambient temperature (21 ± 1°C) and humidity (50 ± 5%) in the animal core facility of Huazhong University of Science and Technology.

### Model of tMCAO and 2,3,5-Triphenyl Tetrazolium Chloride (TTC) Staining

To produce ischemic stroke, we utilized the tMCAO model according to a previous study (Ansari et al., 2011). In brief, 4-month-old C57BL/6J mice were anesthetized with 2% isoflurane using an anesthetic mask with oxygen/air mixture in a stereotactic frame (Stoelting, Wood Dale, IL, USA) to maintain 1% isoflurane. The rectal temperature was maintained at 37°C ± 0.5°C with a constant temperature blanket (Harvard Apparatus, Cambridge, MA, USA) during surgery. Next, a 7/0 surgical nylon monofilament with a rounded tip was introduced into the left internal carotid through the external carotid stump. Laser Doppler flowmetry was applied to monitor the changes of cerebral blood flow and determine the position of monofilament. The right position of monofilament was confirmed by a concurrent drop [value as a percentage (≥80%) relative to baseline] in cerebral blood flow, which stands for the success of tMCAO model. The filaments were kept in place for 60 min (occlusion) and then removed (refill), whereas the control mice were treated similarly except that the middle cerebral artery was not occluded after the neck incision. After mice regained full consciousness, neurological deficits were assessed using a simple five-point scale according to a previous study (Longa et al., 1989).

To determine cerebral infarction and ischemic areas, we stained brain tissues with the TTC stain as described in a previous study (Ansari et al., 2011). Following intraperitoneal injection of pentobarbital-phenytoin solution to execute euthanasia after completion of the experiments (Boivin et al., 2017), the mouse brains were removed after completion of our experiments, rapidly frozen at −20°C for 5 min, prepared for coronal sectioning (seven 1-mm sections *per* mouse), and stained with 2% TTC solution (Cat. #: 298-96-4; Sigma-Aldrich, St. Louis, MO, USA) at 37°C for 20 min. Subsequently, brain sections were reviewed under a stereoscope (Guilin Guiguang Instrument Co., Ltd., Guangxi, China) for the presence and size of infarctions (positive vs. negative TTC staining).

### RNA and miRNA Isolation and Construction of Total and Small RNA-Seq Libraries

Total RNA and miRNA were isolated from mouse brain tissues using an RNAzol^®^ RT RNA Isolation Reagent kit (Sigma-Aldrich) according to the manufacturer’s protocol. In brief, the ischemic core region of mouse brain cortex tissues (50 mg each) were ground in frozen mortar/liquid nitrogen and then transferred into a 2-ml centrifuge tube containing 1 ml of RNAzol^®^ RT for RNA and miRNA isolation. After quantification using the QubitTM RNA HS Assay Kit (Cat. #Q32852; Invitrogen, Carlsbad, CA, USA), these RNA samples were subjected to RNA integrity testing using the Experion RNA StdSens Analysis Kit (Cat. #7007103; Bio-Rad, Hercules, CA, USA) and used for total RNA-Seq library construction using the TruSeq RNA Sample Preparation Kit v2-Set A (Cat. #RS-122-2001; Illumina, San Diego, CA, USA) and TruSeq RNA Sample Preparation Kit v2-Set B (Cat. #RS-122-2002; Illumina), whereas the small RNA-Seq library was prepared using the TruSeq^®^ Small RNA Library Preparation Kits (Cat. # RS-200-0012, #RS-200-0024, #RS-200-0036, and #RS-200-0048; Illumina) following the manufacturer’s instructions. RNA samples with RNA integrity number >8 were used for library preparation. Starting material of 100 ng of total RNA was rRNA depleted followed by enzymatic fragmentation, cDNA synthesis, and double-stranded cDNA purification (AMPure XP, Beckman Coulter, USA). Each RNA-Seq or small RNA-Seq group contains three biological replications.

### Sequencing and Bioinformatics Analysis

Following library preparation, the RNA-Seq libraries were subjected to sequence analysis. Index-encoded samples were prepared *via* the cBot Cluster Generation System (Illumina) using the HiSeq PE Cluster Kit v4 (Cat. #401-4001; Illumina), and then 150-bp pair-end (PE150) RNA-Seq was performed on the HiSeq X platform, whereas 50-bp single-end (SE50) small RNA-Seq was performed on the HiSeq 2500 platform (both from Illumina). The raw data were analyzed for sequencing quality, length distribution of the reads, and adapter contamination using FastQC as recommended by the developer (http://www.bioinformatics.babraham.ac.uk/projects/fastqc). The cutadapt function was used to remove the adapters, short sequences, and the low-quality sequences (Martin 2011). The statistical data of the clean reads that passed through the quality control are listed in **Table 1**. We next analyzed the Clean Read RNA-Seq data using the Subread-FeatureCounts-DESeq2 workflow in hppRNA (Wang, 2018) and mapped them against the latest mouse reference genome sequence GRCm38.p6 (https://www.gencodegenes.org/mouse/) using Subread (Liao et al., 2013). We then counted all expressed genes using FeatureCounts (Liao et al., 2014) and bioinformatically compared and normalized them using DESeq2 (Conesa et al., 2016), one of the most reliable methods to normalize the level of each DE gene.

**Table 1:** Basic information of the sequenced sample data.

Sample_name	RNA_RQI	smallRNA-Seq_clean_reads	RNA-Seq_clean_reads
NC01R1	9.6	17,731,737	131,417,424
NC01R2	9.4	17,160,502	152,761,736
NC01R3	9.5	15,004,999	161,623,258
NC03R1	9.7	46,110,230	154,387,366
NC03R2	10	21,980,066	152,080,990
NC03R3	9.5	18,448,955	150,211,120
NC07R1	9.8	18,795,980	133,329,410
NC07R2	9.5	21,753,041	176,385,470
NC07R3	9.6	16,130,130	139,356,522
NC14R1	9.7	18,628,299	138,656,436
NC14R2	9.4	18,682,679	161,505,522
NC14R3	9.9	16,951,551	160,132,972
NC28R1	10	20,760,411	148,152,290
NC28R2	9.3	26,243,403	138,897,104
NC28R3	9.2	19,476,526	181,555,252
SC01R1	9.5	29,950,281	151,527,848
SC01R2	9.6	25,414,386	151,070,176
SC01R3	9.6	26,953,746	165,881,914
SC03R1	9.2	24,636,125	159,735,374
SC03R2	8.5	25,357,330	147,253,844
SC03R3	9.1	17,836,966	170,407,598
SC07R1	9.8	19,696,891	178,364,232
SC07R2	9.7	27,231,234	164,376,752
SC07R3	9.9	26,808,297	185,886,506
SC14R1	10	17,978,089	160,705,286
SC14R2	9.6	21,156,859	140,030,994
SC14R3	9.5	20,600,385	147,363,924
SC28R1	9.4	18,166,688	163,534,926
SC28R2	9.3	16,858,757	181,231,614
SC28R3	9.3	29,934,724	135,746,966

The RNA quality indicator (RQI) value represents the integrity of the RNA samples as obtained from the Experion automated electrophoresis station. This value is similar with the RIN; e.g., an RNA sample with RQI > 8 could indicate RNA integrity. SNC, stroke vs. non-stroke (control) cortex; 01, 03, 07, 14, 28, day 1, 3, 7, 14, and 28 after tMCAO surgery, respectively; R1, R2, and R3, biological repeat 1, 2, and 3.

To assess intra- and inter-group differences of these RNA-Seq data, we utilized the standardized data sets for principal component analysis (PCA; **Supplementary Figure 1**) and found that the difference between groups at each time point was relatively large, whereas the internal difference was small, indicating that subsequent differential analysis of gene expression was justified. Thus, we performed DE gene analysis by assessing the output counts of the FeatureCounts as the input to DESeq2 and obtained DEgenes for up- and down-regulation at each time point (**Supplementary Table 1**).

For the small RNA-Seq data, we aligned the fastq file to the latest mouse reference genomic sequence GRCm38.p6 using miRDeep2 (Friedlander et al., 2012) to identify known and unknown miRNAs. We then utilized DESeq2 to normalize the level of known miRNAs and performed PCA using the standardized data set to assess the intra- and inter-group differences (**Supplementary Figure 1**). The data showed that the difference between the groups was large, whereas the within-group differences were small at each time point, indicating that the subsequent differential analysis of gene expression was justified. We therefore identified DEmiRNAs for up- and down-regulation at each time point (**Supplementary Table 2**) using DESeq2 followed by miRWalk3.0 (Dweep et al., 2011; Dweep and Gretz, 2015) with default arguments to predict target genes for DEmiRNAs.

To identify time-dependent DEmiRNAs, we used the maSigPro package (Conesa et al., 2006) with default parameters. Gene ontology (GO) enrichment was also assessed using the enrichGO functions in R package clusterProfiler (version 3.6.0) (Yu et al., 2012) and the significance of the enriched GO terms was evaluated using a hypergeometric test with a false discovery rate <0.05. Our sequencing data have been uploaded to the Genome Sequence Archive with accession numbers CRA001143 and CRA001432.

### Quantitative Reverse Transcription-Polymerase Chain Reaction (RT-qPCR)

Total miRNA was isolated using an RNAzol^®^ RT RNA Isolation Reagent kit (Sigma-Aldrich) according to the manufacturer’s protocol and reverse transcribed into cDNA by using the All in one First strand cDNA Synthesis kit reverse transcription reagent (Cat. #AORT-0050; GeneCopoeia, Rockville, MD, USA) according to the manufacturer’s protocols. These cDNA samples were then amplified using qPCR with different miRNA primers purchased from Tiangen Biochemical Technology Co., Ltd. (Beijing. China) without disclosure of the proprietary primer sequences. The reactions were set up in duplicate in total volumes of 10 μl containing 5 μl of 2× miScript SYBR green PCR mix (Cat. # 218073; GeneCopoeia) and 2 μl of template (1:5 dilution from RT product) with a final concentration of 400 nM of the primer. The PCR was performed using a real-time PCR instrument (Cat. # 1855201; Bio-Rad) with the PCR cycle as follows: 95°C/3 min, 40 cycles of 95°C/30 s, 60°C/45 s, and 95°C/1 min, followed by melt-curve analysis to verify that a single product *per* primer pair was amplified, which was quantified using the 2(−ΔΔct) method. Each qRT-PCR group has three biological replications.

## Results

### Differential Expression of the Transcriptome at Different Stages of Ischemic Stroke

**Figure 1:**
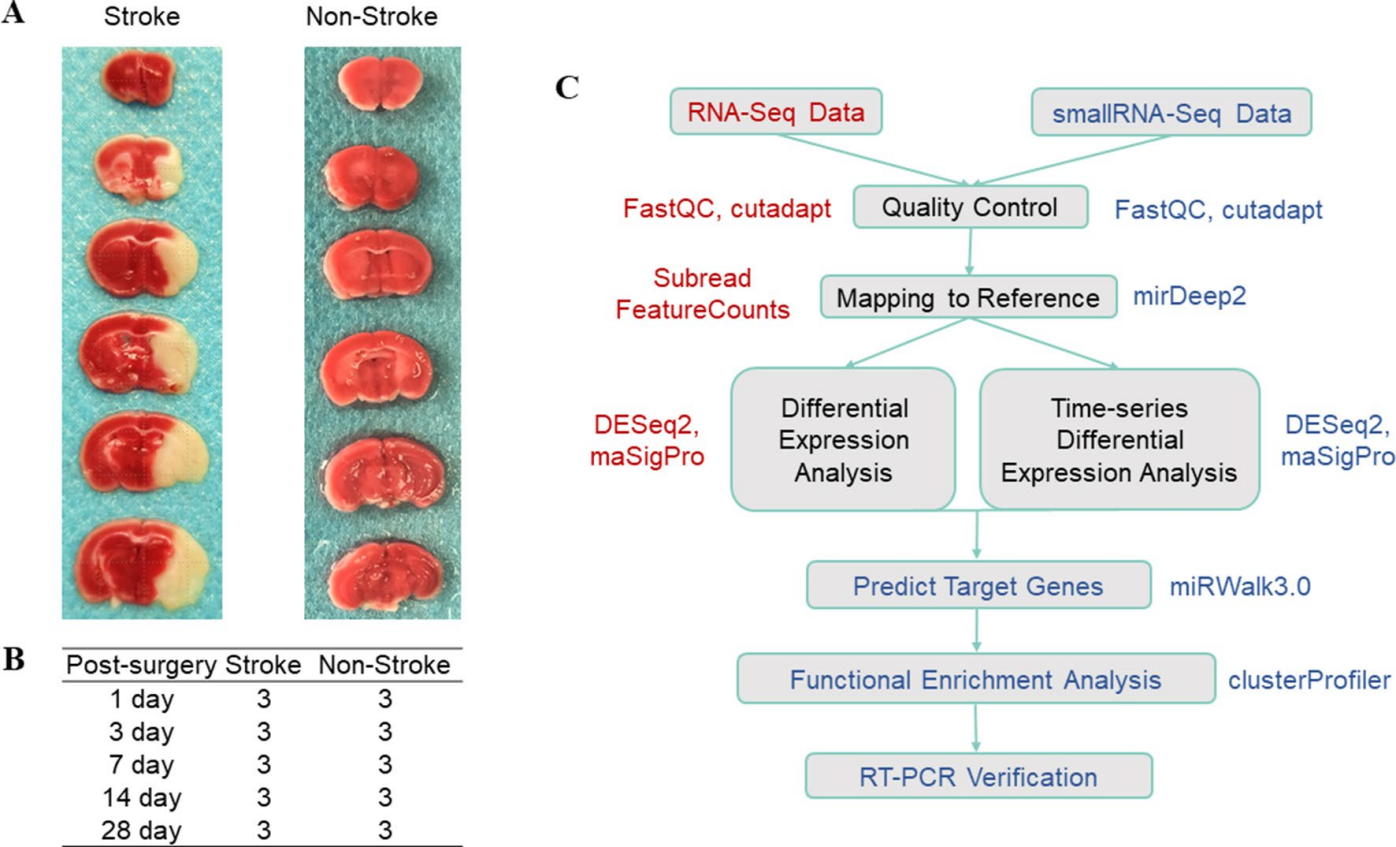
Illustration of the data composition and data analysis process. **(A)** TTC staining after tMCAO; the white part indicates the ischemic region. **(B)** Experimental design. The stroke group indicates the group of mice that underwent tMCAO surgery, whereas the non-stroke group is the sham operation group. The experiments were performed in triplicate at each time point. **(C)** Data analysis work flow. The box shows the specific steps, the color text next to the box indicates the software used in the step, whereas the red color indicates RNA-Seq data and the blue color indicates small RNA-Seq data.

In this study, we used a mouse model of tMCAO with an occlusion time of 1 h in 4-month-old C57BL/6J mice and then removed ischemic cortex tissues on days 1, 3, 7, 14, and 28 (*n* = 3; **Figures 1A, B**). We then constructed and sequenced 150-bp pair-end (PE150) RNA-Seq libraries and 50-bp single-end (SE50) small RNA-Seq libraries. After quality control of the data (**Supplementary Figure 1** and **Table 1**), our sequencing data analysis (**Figure 1C**) identified DEgenes and DEmiRNAs at each time point (**Supplementary Tables 1** and **2**).

### Inverse Association of DEmiRNAs With DEgenes at Each Time Point of Ischemic Stroke and Their Functional Enrichment

**Figure 2:**
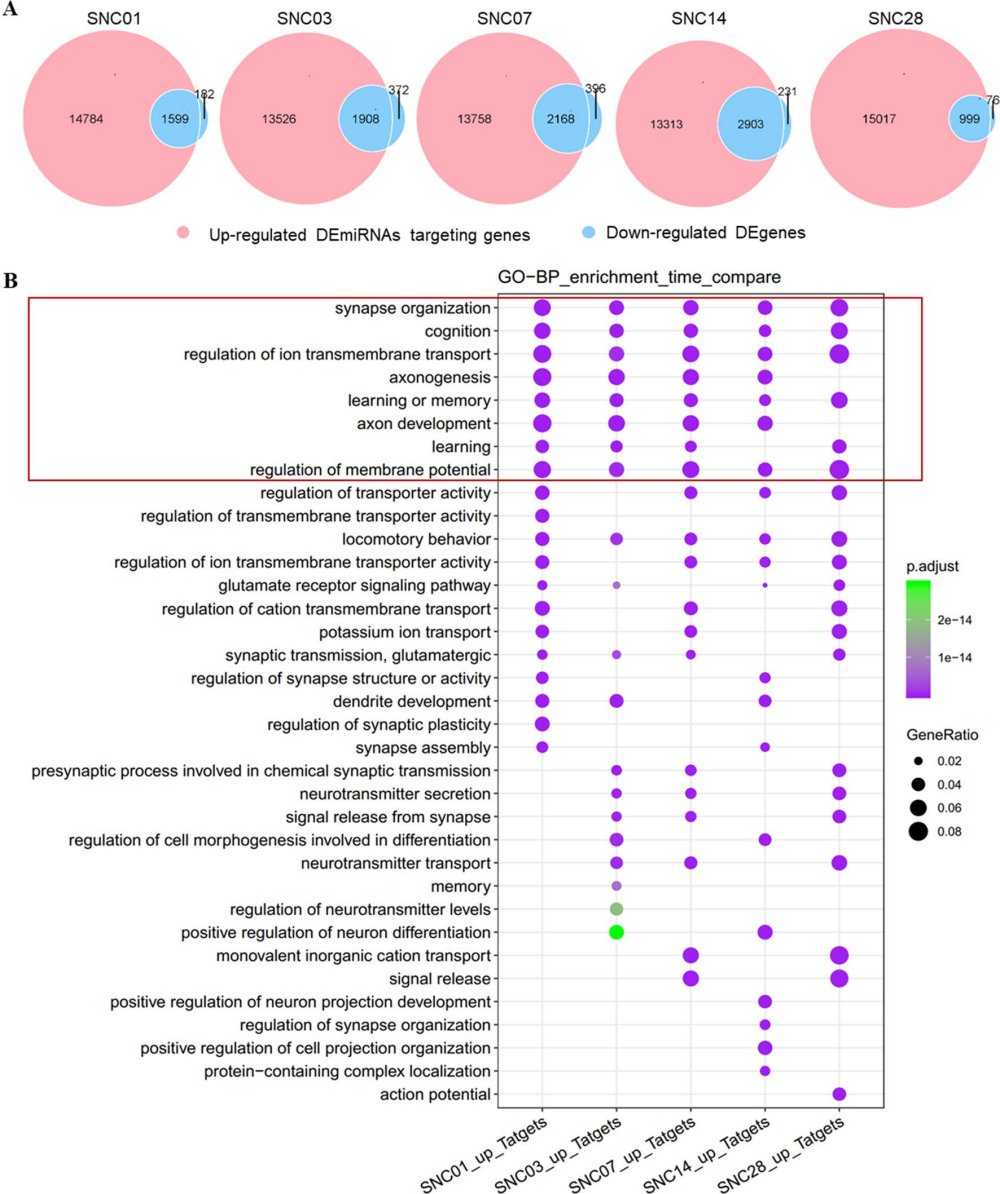
Overlap of up-regulated DEmiRNAs vs. down-regulated DEgenes at different stages of ischemic stroke and their functional enrichment. **(A)** Venn diagram of up-regulated DEmiRNAs vs. the down-regulated DEgenes at different stages of ischemic stroke. Red represents up-regulated DEmiRNAs whereas blue represents the down-regulated DEgenes at the given time point. SNC, stroke vs. non-stroke (control) cortex; 01, 03, 07, 14, and 28, days 1, 3, 7, 14, and 28 after tMCAO surgery, respectively. **(B)** Gene sets at the intersection of each time point for the GO enrichment. An example comparison of the top 20 biological process (BP) pathways at each time point and the size of the point represents the proportion of the number of genes enriched in a given pathway in the background, whereas the background of the genes at each time point is the input of all the gene sets at that time point. The color of the dots represents the *p* value and the purple to green gradient shows the statistical significance from the strong to weak values.

We performed bioinformatics analysis of these DEmiRNAs, associated up-regulated DEmiRNAs with down-regulated DEgenes (Chen and Boutros, 2011) at each time point of ischemic stroke, and analyzed enrichment of functional categories of these DEgenes. We observed an intersection of DEgene down-regulation with DEmiRNA up-regulation at each time point (**Figure 2A**). Approximately 90% of the down-regulated DEgenes overlapped with up-regulated DEmiRNAs targeting these genes. Furthermore, gene functional enrichment analysis (Yu et al., 2012) revealed a number of DEgenes altered across all time points (**Figure 2B**) that were enriched in biological pathways related to neuronal function such as the synapses, cognition, axonogenesis, and ion transmembrane transport. These data revealed robust changes in gene expression after ischemia stroke that were predicted to impact neuronal function.

**Figure 3:**
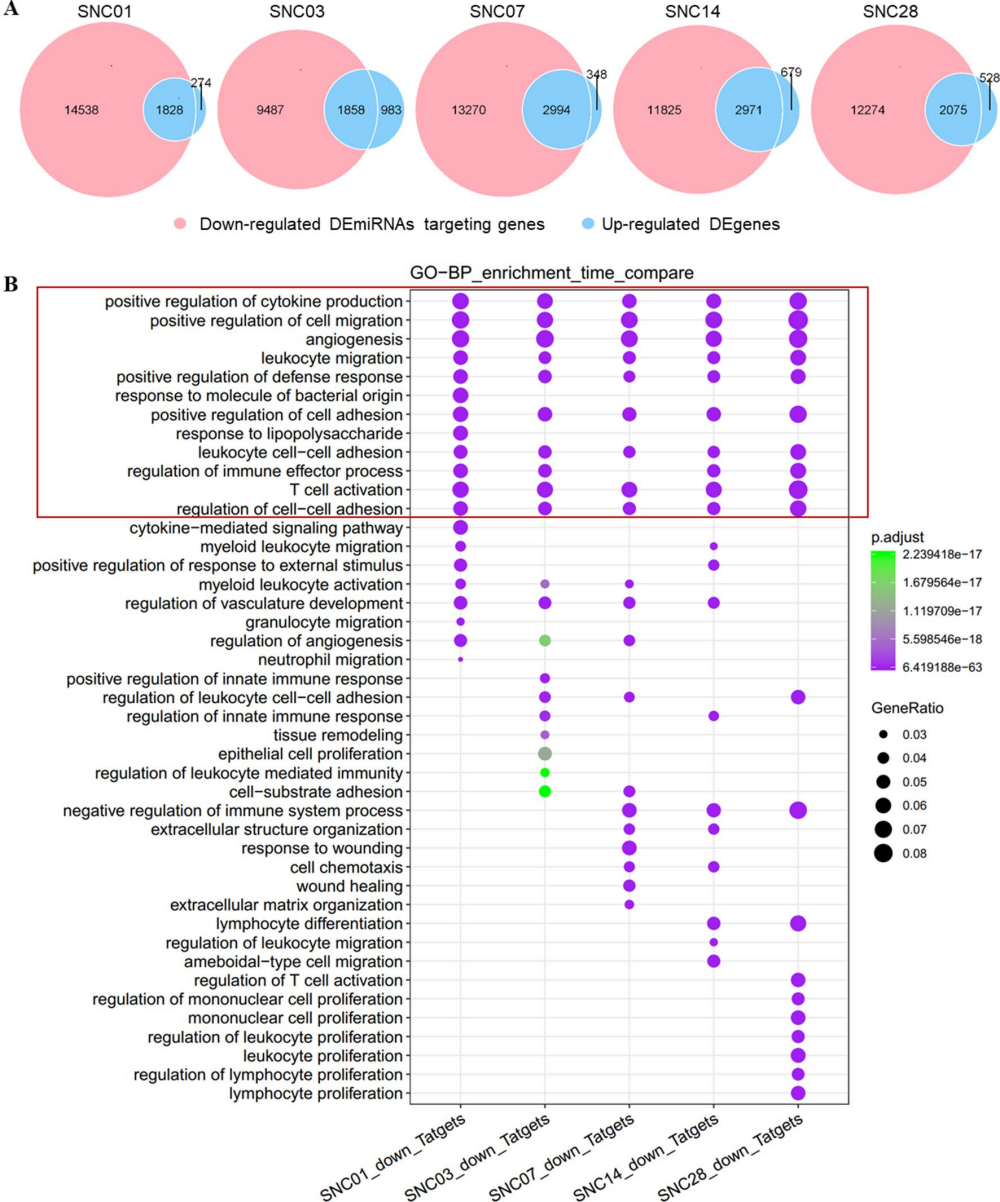
Overlap of down-regulated DEmiRNAs targeting genes vs. the up-regulated DEgenes at different stages of ischemic stroke and their functional enrichment. **(A)** Venn diagram of the down-regulated DEmiRNAs vs. the up-regulated DEgenes at different stages of ischemic stroke. Red represents the down-regulated DEmiRNAs, whereas blue represents up-regulated DEgenes at the same time point. SNC, stroke vs. non-stroke (control) cortex; 01, 03, 07, 14, and 28, days 1, 3, 7, 14, and 28 after tMCAO surgery, respectively. **(B)** Gene sets in the intersection of each time point for the GO biological process (BP) function enrichment. An example comparison of the top 20 pathways at each time point; the size of the point represents the proportion of the number of genes enriched in a given pathway in the background, whereas the background of the gene at each time point is the input of all the gene sets at that time point. The color of the dots represents *p* value, whereas the purple to green gradient shows the statistical significance from the strong to weak values.

Similarly, we associated down-regulated DEmiRNAs with up-regulated DEgenes at different stages of ischemic stroke and performed functional enrichment analyses. Our data showed an association of these down-regulated DEmiRNAs with the up-regulated DEgenes at each time point of stroke (**Figure 3A**). The functional enrichment analysis showed that up-regulated gene sets were enriched in pathways such as immune responses (e.g., cytokine production), cell adhesion, and immune cell migration and angiogenesis (**Figure 3B**), suggesting the potential activation of angiogenesis and inflammatory pathways in the brain after stroke, which was consistent with previous findings (Barone and Feuerstein, 1999; Li et al., 2015; Yin et al., 2015).

We also observed activation of discrete biological pathways in up-regulated DEgenes at each time point after stroke. For example, on day 7, the injury repair pathway was activated, whereas on day 28, pathways associated with cell proliferation were activated. These data demonstrated a change in distinct neuronal repair and proliferation-associated genes at different stages following ischemic injury.

### Time-Dependent Changes in DEmiRNAs

**Figure 4:**
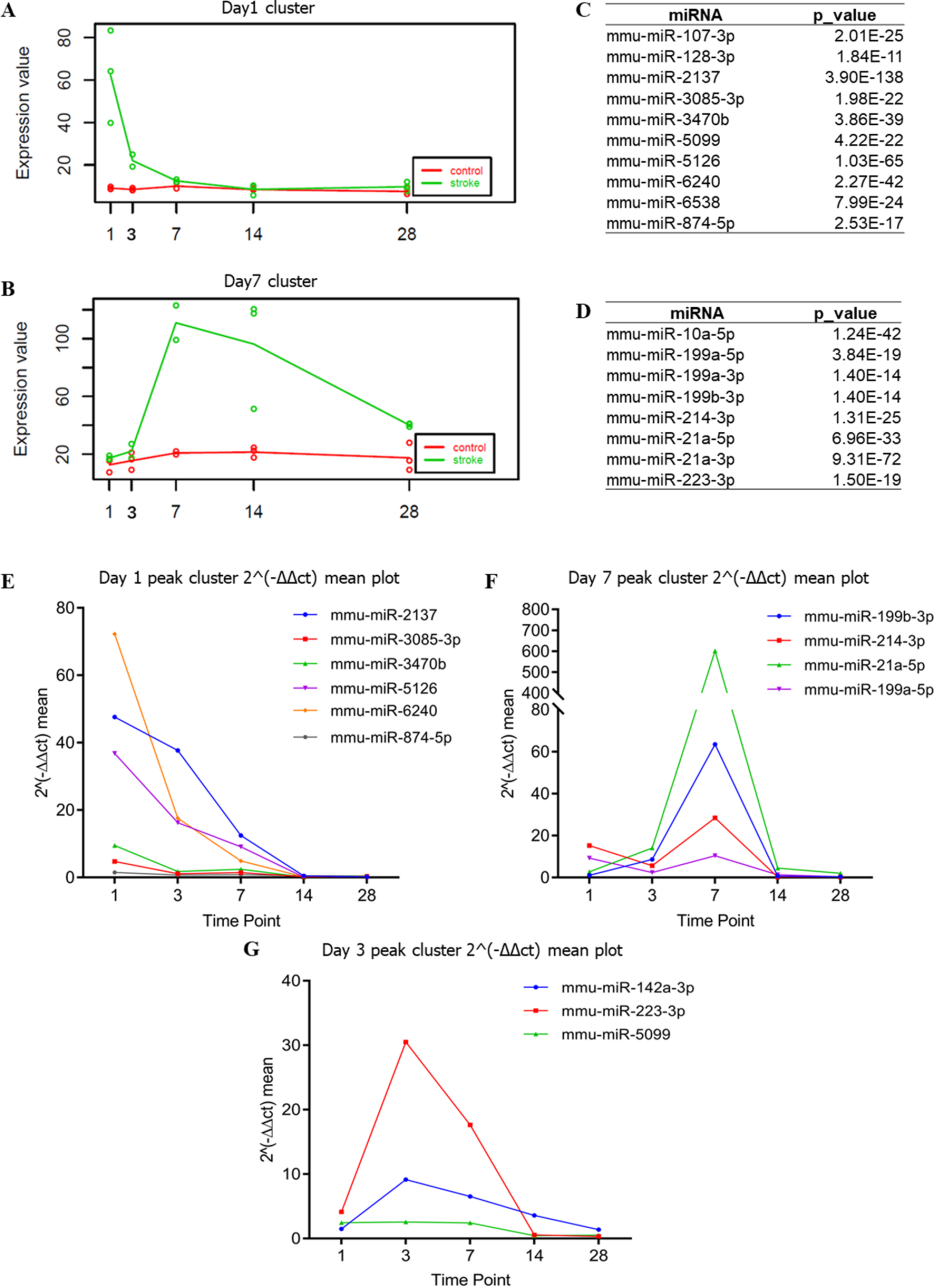
Time-dependent changes in DEmiRNAs. **(A**, **B)** Time-dependent DEmiRNAs identified by maSigPro. The abscissa indicates different time points, whereas the ordinate indicates the normalized expression. The green line indicates the stroke group, whereas the red line indicates the non-stroke (control) group. **(C**, **D)** Table summarizing the DEmiRNAs enriched with a trend consistent to that of **(A)** and **(B)**, respectively. **(E**–**G)** Time-dependent DEmiRNAs identified by RT-qPCR. The abscissa is the time point, whereas the ordinate is the mean expression of the three biological replicates. RT-qPCR used the value of 2−ΔΔct to indicate the expression of each DEmiRNA.

**Figure 5:**
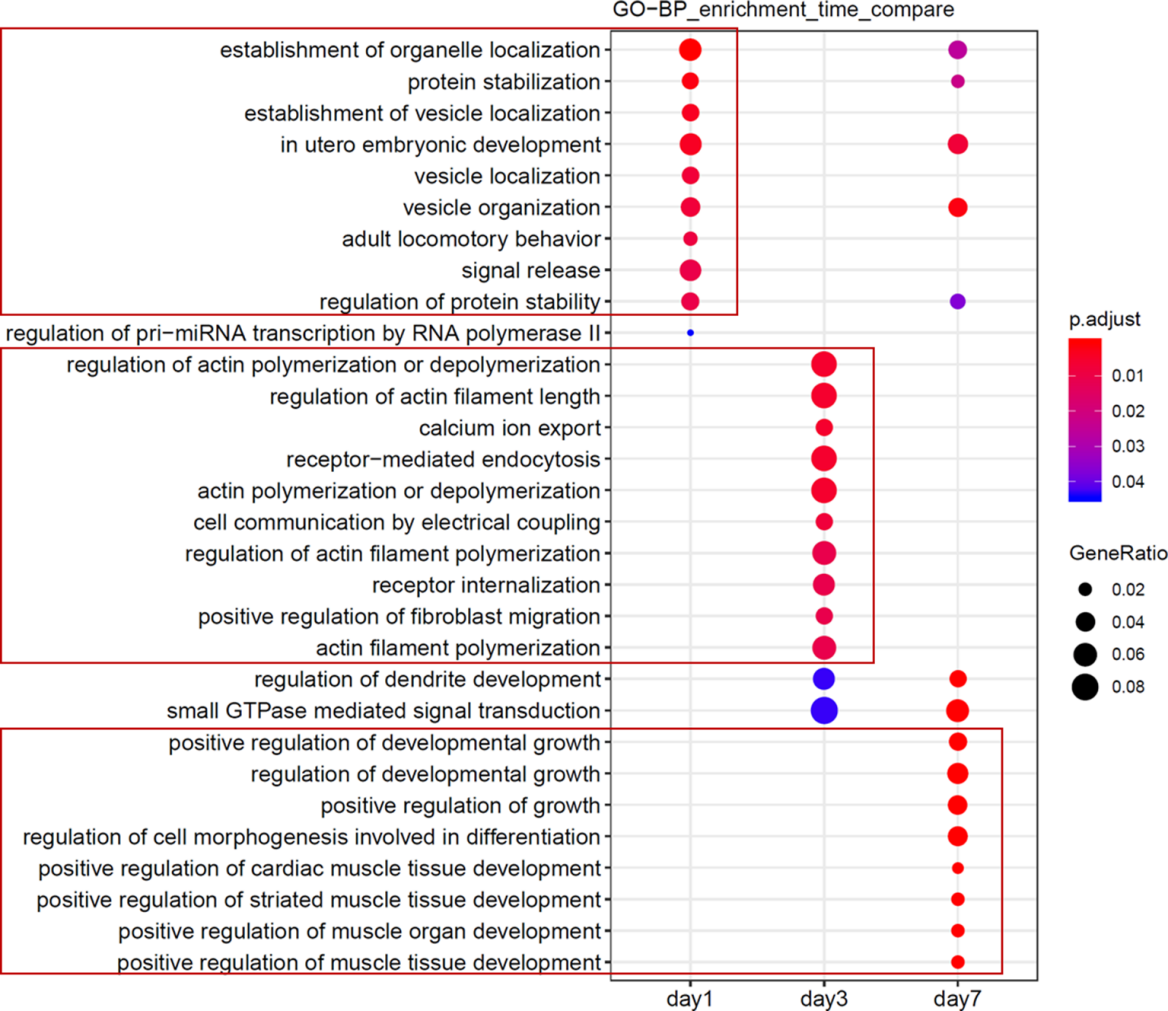
Functional analyses of candidate miRNAs. Functional enrichment comparison of gene targets of miRNAs in **Figure 4A and B**, with the abscissa representing the cluster of days, and the ordinate being the entry of the GO annotation.

Next, we assessed and identified the time-dependent changes in these DEmiRNAs using the maSigPro package (Conesa et al., 2006). We found 28 time-dependent DEmiRNAs (**Supplementary Table 3**), several of which displayed enrichment at specific time points (**Figures 4A–D**, **Supplementary Figure 2**). Functional enrichment analysis of these DEmiRNAs’ targets showed that the most significant GO terms at day 1 included signal transduction and autophagy, and day 3 included regulation of actin filament polymerization, whereas day 7 peak DEmiRNAs included those involved in dendrite development, cell migration, and angiogenesis (**Figure 5**). We verified expression of these 28 time-dependent DEmiRNAs in mouse brain tissues after ischemic stroke using RT-qPCR. Consistent with the small RNA-Seq data, these miRNAs could be divided into three clusters with peak expression levels on days 1, 3, and 7 (**Figures 4E–G**).

Following cerebral ischemia–reperfusion (equivalent to thrombolytic therapy after human stroke), the expression levels of a group of miRNAs (mmu-miR-2137, mmu-miR-3085-3p, mmu-miR-3470b, mmu-miR-5126, mmu-miR-6240, and mmu-miR-847-5p) was reduced over time (**Figure 4E**), whereas other miRNAs (mmu-miR-21-5p, mmu-miR-199b-3p, mmu-miR-199a-5p, and mmu-miR-214-3p) were up-regulated at peak levels 7 days after cerebral ischemia–reperfusion (**Figure 4F**). We also identified a novel group of miRNAs including mmu-miR-223-3p, mmu-miR-142a-3p, and mmu-miR-5099 (**Figure 4G**) that were up-regulated 3 days after cerebral ischemia–reperfusion.

### Functional Enrichment of Time-Dependent DEmiRNAs

**Figure 6:**
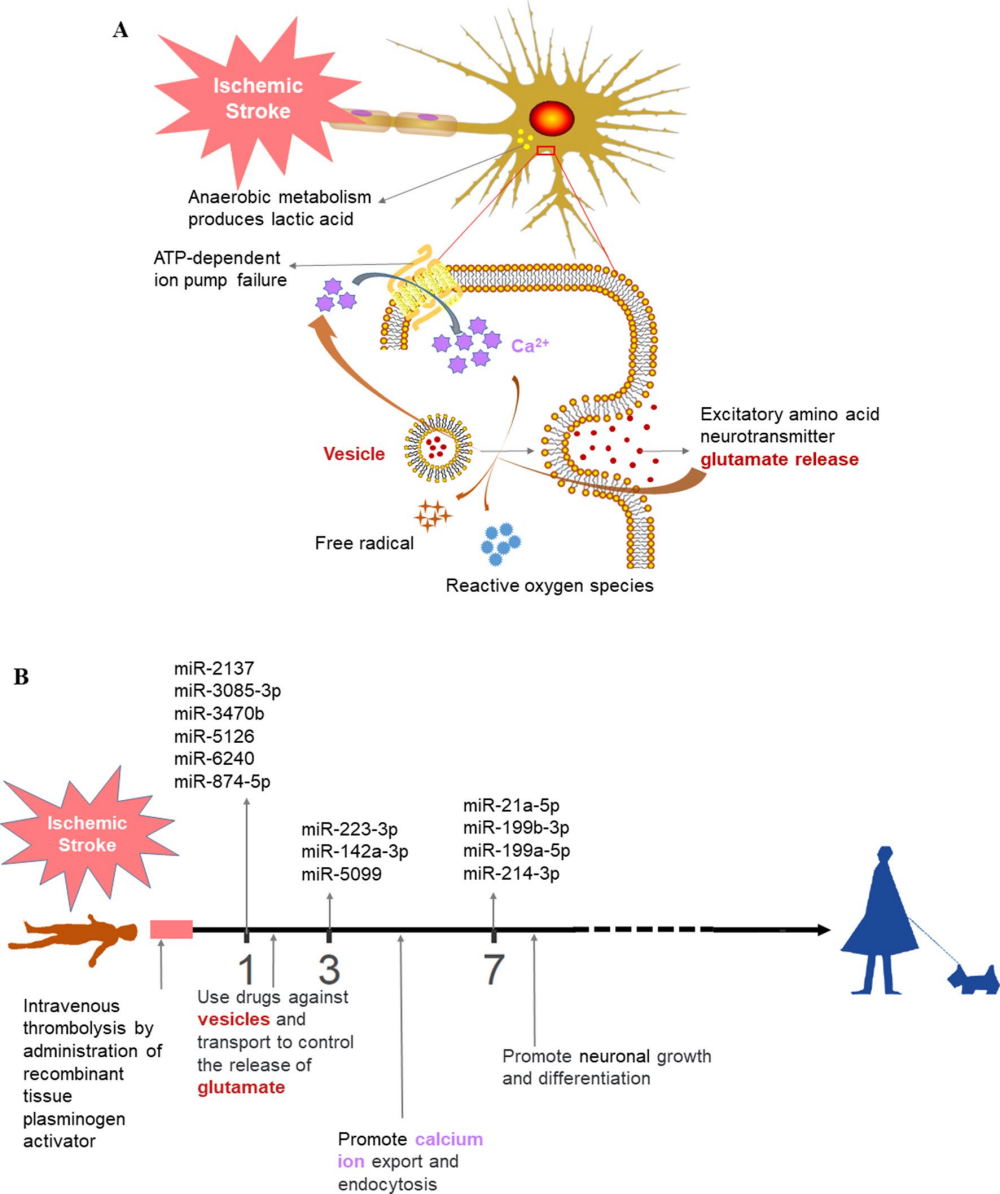
Prospective ischemic stroke treatment. **(A)** Schematic diagram of the pathological mechanism of ischemic stroke. **(B)** Timeline illustrating the future treatment of ischemic stroke. The colors of the text are consistent between **(A)** and **(B)**.

We then performed functional enrichment of time-dependent DEmiRNAs for potential biomarker discovery using Targetscan (Agarwal et al., 2015), miRDB (Wang, 2008; Wang and El Naqa, 2008; Wong and Wang, 2015; Wang, 2016), and miRT (Bhattacharyya et al., 2012) databases (**Figure 5**). Following ischemic stroke (**Figure 6A**), hypoxia causes ATP depletion, leading to failure of the Na^+^/K^+^ pump, neuronal depolarization, and glutamate release. If glutamate cannot be absorbed by the neurons or astrocytes, glutamate levels could rise rapidly in the extracellular space of the brain, resulting in activation of glutamate receptors, mainly *N*-methyl-d-aspartate receptor, to induce Ca^2+^ into cells and, in turn, activation of calpain, phospholipase, and neuronal nitric oxide synthase and production of reactive oxygen species to promote cell death (Schinder et al., 1996; Rossi et al., 2000; Broughton et al., 2009; Fricker et al., 2018). To date, several types of cell death have been associated with excitotoxicity, such as apoptosis, autophagy, and phagocytosis.

Our data confirmed that functions of target genes during the early stage of ischemic stroke recovery (day 1) were concentrated in vesicle production, transport, and release. Functional enrichment of target genes on day 3 after ischemic stroke was concentrated in calcium ion export, endocytosis, cell communication by electrical coupling, and fibroblast migration. Lastly, functional enrichment of target genes on day 7 after ischemic stroke was mainly concentrated in cellular development, growth, and differentiation. Thus, the three groups of miRNAs may represent biomarkers corresponding to each time point after ischemic stroke; i.e., mmu-miR-2137, mmu-miR-3085-3p, mmu-miR-3470b, mmu-miR-5126, mmu-miR-6240, and mmu-miR-847-5p (for day 1); mmu-miR-223-3p, mmu-miR-142a-3p, and mmu-miR-5099 (day 3); and mmu-miR-21-5p, mmu-miR-199b-3p, mmu-miR-199a-5p, and mmu-miR-214-3p (day 7).

## Discussion

In the current study, we used a mouse model of tMCAO to obtain brain tissues for RNA isolation, RNA-Seq, small RNA-Seq library construction, sequencing, and bioinformatics analysis. We identified groups of DEgenes and DEmiRNAs at various time points from 1 to 28 days after stroke. Our bioinformatics analysis data showed significant associations of up-regulated DEmiRNAs with down-regulated DEgenes at each time point after ischemic stroke. Conversely, down-regulated DEmiRNAs were associated with up-regulated DEgenes at different stages of ischemic stroke. Down-regulated DEgenes were enriched in biological pathways of neuronal function such as synapses, cognition, axonogenesis, and ion transmembrane transport, whereas up-regulated DEgenes were enriched in functional terms including immune responses and angiogenesis. We also revealed the activation of different biological pathways at different time points after stroke and identified three groups of miRNAs corresponding to days 1, 3, and 7 after ischemic stroke. Our findings support these novel three groups of miRNAs as molecules warranting further investigation as biomarkers to assess the stages of ischemic stroke recovery and treatment.

It is known that cerebral ischemia–reperfusion injury induces a complex pathophysiological cascade that includes a wide range of aberrant cellular processes. In the ischemic phase, reduced blood supply rapidly leads to failure of ionic, gradients, excitotoxicity, and neuronal death. During the reperfusion phase, the return of oxygen contributes to oxidative stress, and the restoration of blood introduces factors that promote inflammation and edema, thereby further increasing the vulnerability of the affected tissue to neurodegeneration. The expression levels of hundreds of miRNAs were shown to be altered after transient focal ischemia after as early as 30 min and as late as 7 days of reperfusion. We currently observed that several miRNAs rapidly respond to focal ischemia and their expression changes by a very high magnitude. Furthermore, the ischemia-induced miRNA changes sustain for at least up to 7 days of reperfusion. We presume that the effect of focal ischemia seems to be greater on the genes that transcribe miRNAs than those transcribe protein coding RNAs. In vertebrates, many miRNA genes are in the intragenetic regions and the introns of the coding regions. Ischemia can independently influence the transcription of mRNAs and miRNAs. Although, the mechanisms that regulate miRNA transcription after focal ischemia are still unknown, changes in the miRNA synthetic RNases (Dicer and Drosha) after stroke may influence the expression profiles and lead to the difference of miRNAs at different stages of ischemic stroke. It is also known that certain mRNAs and/or their protein products may also control the expression of specific miRNAs. In this study, we used RNA-Seq, small RNA-Seq, and qRT-PCR, which improved our ability to detect more comprehensive alterations following experimental stroke. We identified a group of DEgenes and DEmiRNAs at each time point after stroke and found an association of up-regulated DEmiRNAs with their targeted down-regulated DEgenes, whereas conversely down-regulated DEmiRNAs were associated with up-regulated targeted DEgenes at different stages of ischemic stroke. Our data are consistent with the function of miRNAs to regulate the expression of protein-coding genes, and our results regarding the differential expression of miRNA-4639, miRNA-146a, miRNA-181b, miRNA-124, and miRNA-128 are consistent with previous studies (Beveridge et al., 2008; Wang et al., 2013; Arena et al., 2017; Chen et al., 2017). We also identified numerous novel miRNAs related to ischemic stroke, such as miR-2137, miR-874-5p, and miR-5099, although further study is needed to verify their role in this disorder. Furthermore, we linked these DEmiRNAs to their potential gene targets, with functional enrichment analysis revealing that down-regulated DEgenes were enriched in biological pathways of neuronal function such as synapses, cognition, axonogenesis, and ion transmembrane transport. These findings are supported by previous studies (Kristian and Siesjo, 1996; Terasaki et al., 2014; Deleglise et al., 2018) and suggested that neuronal function following ischemia stroke is severely affected. In addition, the up-regulated DEgenes included pathways of immune responses and angiogenesis, which are also consistent with previous studies (Barone and Feuerstein, 1999; Li et al., 2015; Yin et al., 2015) and may reflect activation of the inflammatory response in the brain following stroke. In addition, miRNA–mRNA interaction may be involved in the ischemic stroke. We have performed analysis of the three groups of miRNAs with their targets, and obtain some predicted functions of their interaction (see **Supplementary Figure 3**). By investigating the function of these co-targeted genes (marked by red boxes), we infer the mechanism of the interaction between miRNAs and mRNA in ischemic stroke: a) The mmu-miR-3085-3p and mmu-miR-3085-3p co-regulated Elf1 genes to promote early recovery of ischemic stroke in immune regulation and angiogenesis. b) mmu-miR-214-3p/-199b-3p regulated both Pon2 and Qk to inhibit neuronal apoptosis and play a neuroprotective role in the recovery phase of ischemic stroke. c) mmu-miR-199a-5p and mmu-miR-199b-3p regulated the Taok1 gene to inhibit the inflammatory response during the recovery phase of ischemic stroke and exert neuroprotective effects. However, these predicted functions require further wet experiments to verify. Therefore, future subsequent studies are needed to validate the above functions.

Furthermore, the present study demonstrated that different biological pathways were activated at discrete time points following stroke. For example, on day 7, the injury repair pathway was activated, whereas on day 28, pathways associated with cell proliferation were activated. The proliferation of immune cells can mediate neuronal repair, and the neuroinflammatory response may play an important role in tissue repair (Schwartz and Deczkowska, 2016; Sochocka et al., 2017). Moreover, the present study identified three groups of DEmiRNAs at different stages of ischemic stroke that were verified by RT-qPCR. However, the exact functions of these DEmiRNAs in mediating ischemic stroke development and recovery remain unclear. Further study is needed to explore the expression and function of these miRNAs in models of ischemic stroke. In addition, our current data may have relevance for future treatment of ischemic stroke (**Figure 6B**). Patients suffering from stroke should be monitored for the expression of serum miRNA markers prior to and following thrombolytic therapy, which may inform the treatment with drugs that target neurotransmitter biosynthesis, packaging, and release at day 1. At day 3, in comparison, the miRNA markers might reveal the activation of calcium ion export and endocytosis, with treatment focusing on the reduction of excitatory damage and maintenance of the cellular microenvironment homeostasis. At day 7, treatment may shift to facilitate neuronal repair, growth, and differentiation in order to reduce ischemic stroke-induced mortality and disability and improve the quality of life in patients exhibiting stroke.

The data from our current study constitute a proof of principle that miRNAs may represent attractive biomarkers to assay stroke recovery. In addition, our study provided a large quantity of second-generation raw sequencing data that might be utilized by other scientists, especially by bioinformaticians to generate improved databases in the future to develop more sensitive, specific, and efficient multi-omics data sets.

## Data Availability

The datasets generated for this study can be found in the Genome Sequence Archive with accession numbers CRA001143 and CRA001432.

## Ethics Statement

The animal study was reviewed and approved by Medical Ethics Committee of Tongji Medical College, Huazhong University of Science and Technology.

## Author Contributions

YL and LP conceived and designed the study and prepared the manuscript. YC carried out the high-throughput sequencing and bioinformatics analysis. XH supervised the bioinformatics analyses. YZ, XK, and YG performed animal experiments and RT-qPCR. CY, NT, and PP bred the mice and performed the TTC staining. GX, LF, ZZ, JL, and YF performed statistical analyses; RW completed the data analysis and results summary of the supplementary 2 and 3. All authors participated in the data analysis and approved the final version of this manuscript.

## Funding

This work was supported in part by grants from the National Natural Science Foundation of China (Grant Nos. 31721002 and 91632306 to YL, 81870932 and 81571078 to LP, and 81701282 to RW), the Fundamental Research Funds for the Central Universities (HUST: 2018KFYYXJJ075), and the China Scholarship Council (File No. 201706160023). The Open Access publication charge of this manuscript was provided by the National Natural Science Foundation of China (Grant Nos. 91632306 and 81870932).

## Supplementary Material

Supplementary Figure 1 Principal Component Analysis (PCA). The normalized data set was used for the PCA; the PCA-plots for small RNA-Seq data are shown on the left, whereas those for RNA-Seq data are on the right. From the top to the bottom, the time points indicate 1, 3, 7, 14, and 28 days after tMCAO surgery, respectively. At each time point, the difference between groups is relatively large, whereas the difference within groups is small.

Supplementary Figure 2 Heatmaps. (A) Heatmap for 28 time-dependent DEmiRNAs, we can see that there is almost no difference in the miRNAs in the control group. (B) Heatmap only for the experimental groups using mean value of expression of three biological replicates. Due to interference at the last two time points, differentially expressed miRNAs at various time points cannot be perfectly clustered. (C) Heatmap for miRNAs specifically expressed in day 1, day 3 and day 7.

Supplementary Figure 3 miRNA-mRNA Interaction. (A) Network interactions between biomarkers and their target genes for day 1. (B) Network interactions between biomarkers and their target genes for day 7. No interaction between biomarkers on day 3.

Supplementary Table 1 Excel spreadsheet listing DEgenes at each time point after stroke.

Supplementary Table 2 Excel spreadsheet listing DEmiRNAs at each time-point after stroke.

Supplementary Table 3 List of 28 time-dependent DEmiRNAs identified by maSigPro.

Sequencing data have been uploaded to the Genome Sequence Archive with accession numbers CRA001143 and CRA001432.
