# Supplementary figures and images for: Transcriptome Sequencing Unravels Potential Biomarkers at Different Stages of Cerebral Ischemic Stroke
Source: Front Genet. 2019 Sep 24;10:814. doi: 10.3389/fgene.2019.00814 (PMC6798056; doi:10.3389/fgene.2019.00814)

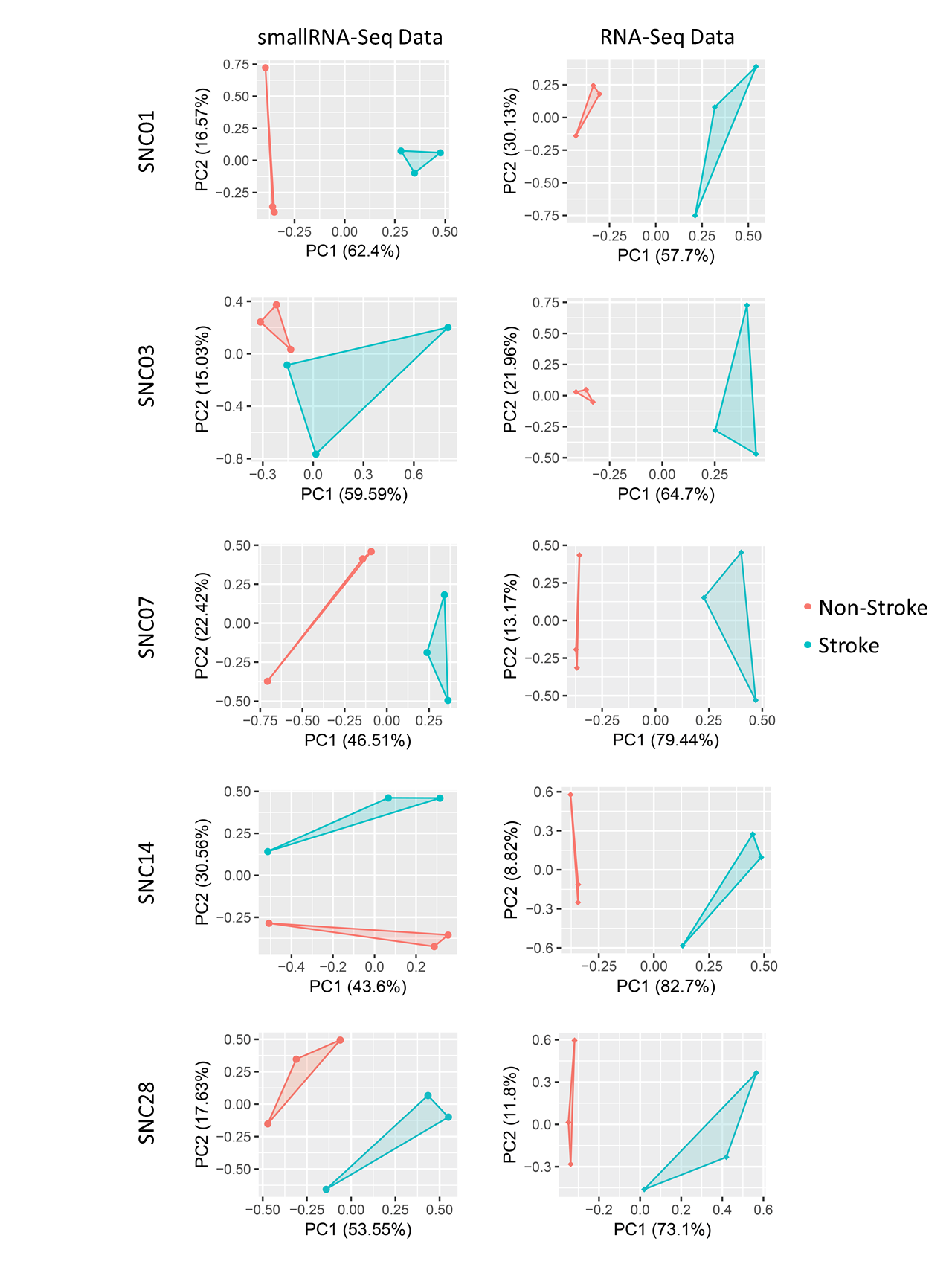

Supplement: Supplementary Figure 1 — Principal Component Analysis (PCA). The normalized data set was used for the PCA; the PCA-plots for small RNA-Seq data are shown on the left, whereas those for RNA-Seq data are on the right. From the top to the bottom, the time points indicate 1, 3, 7, 14, and 28 days after tMCAO surgery, respectively. At each time point, the difference between groups is relatively large, whereas the difference within groups is small. [file Image_1.tif]

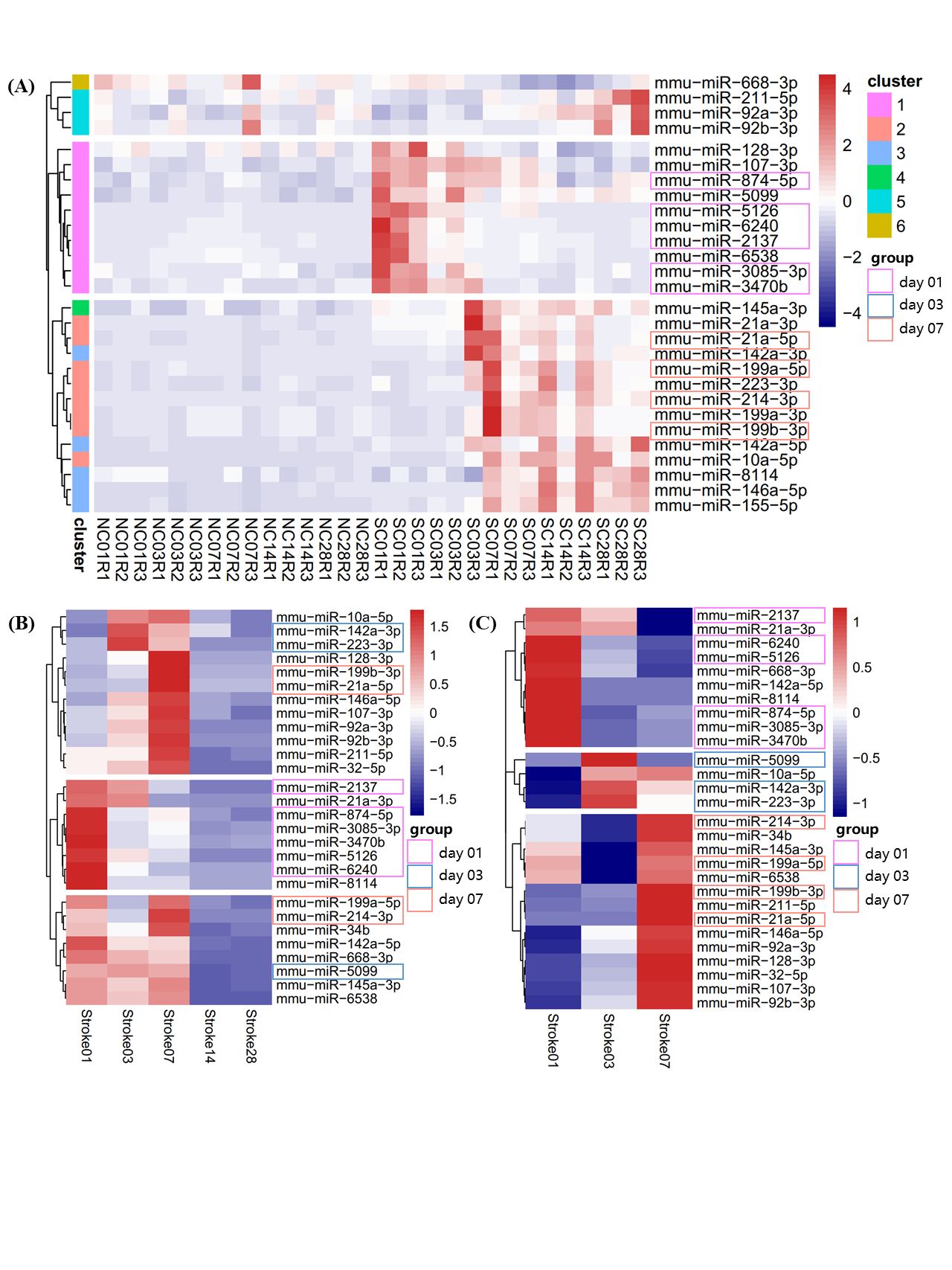

Supplement: Supplementary Figure 2 — Heatmaps. (A) Heatmap for 28 time-dependent DEmiRNAs, we can see that there is almost no difference in the miRNAs in the control group. (B) Heatmap only for the experimental groups using mean value of expression of three biological replicates. Due to interference at the last two time points, differentially expressed miRNAs at various time points cannot be perfectly clustered. (C) Heatmap for miRNAs specifically expressed in day 1, day 3 and day 7. [file Image_2.tif]

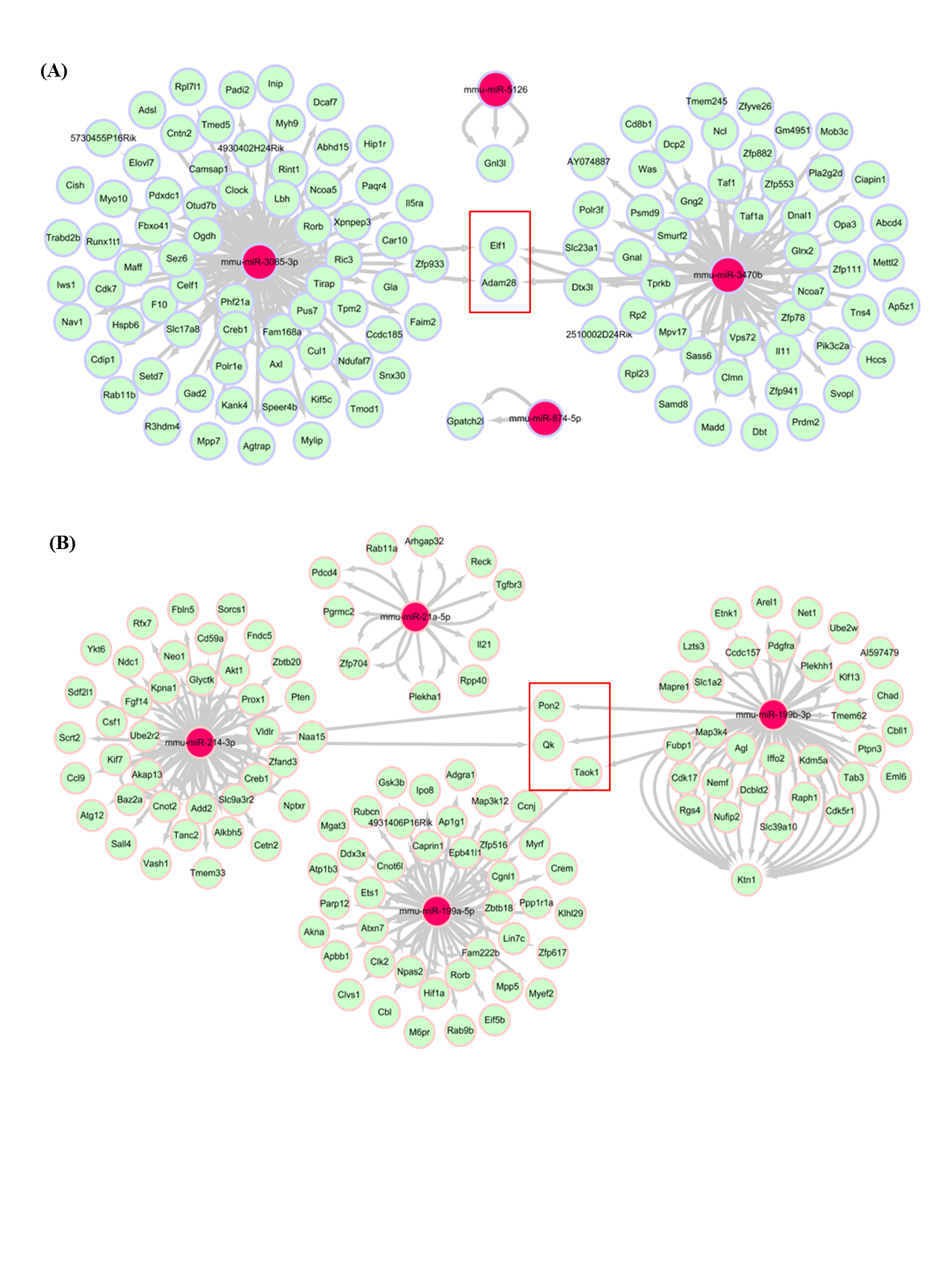

Supplement: Supplementary Figure 3 — miRNA-mRNA Interaction. (A) Network interactions between biomarkers and their target genes for day 1. (B) Network interactions between biomarkers and their target genes for day 7. No interaction between biomarkers on day 3. [file Image_3.tif]
